# Objective and subjective sleep characteristics in hospitalized older adults and their associations to hospital outcomes

**DOI:** 10.3389/frsle.2024.1346642

**Published:** 2024-04-05

**Authors:** Terri L. Blackwell, Sarah C. Robinson, Nicholas Thompson, Lisa Dean-Gilley, Phillip Yu, Alice Pressman, Katie L. Stone

**Affiliations:** ^1^Sutter Health California Pacific Medical Center Research Institute, San Francisco, CA, United States; ^2^Sutter Health, Center for Health Systems Research, Walnut Creek, CA, United States; ^3^Sutter Tracy Community Hospital, Tracy, CA, United States

**Keywords:** actigraphy, acute care, sleep, hospitalization, pilot study

## Abstract

**Purpose:**

Sleep in the hospital setting is understudied, with limited literature describing measurement of sleep quality. This study among older inpatients in an acute-care hospital describes sleep characteristics both objectively and subjectively, and explores the associations of sleep with hospital outcomes.

**Materials and methods:**

Older patients (*n* = 112) at Sutter Tracy Community Hospital were enrolled from January 2016 to November 2017. Sleep prior to admission was measured subjectively [Pittsburgh Sleep Quality Index (PSQI)], while sleep during hospitalization was measured subjectively (sleep diaries) and objectively (actigraphy, averaged over all nights). Outcomes measured included change in cognition during the hospital stay (i.e., Montreal Cognitive Assessment), length of stay (LOS), discharge to a skilled nursing facility (SNF), and re-admittance to a hospital within 30 days of discharge.

**Results:**

The participants were on average 68.7 ± 6.5 years old, predominately white (77%) and 55% women. Average PSQI was high (9.1 ± 4.2) indicating poor sleep quality prior to admission. Actigraphy was well-tolerated, with most (89%) having complete data. Sleep during the hospital stay was disturbed, with low levels of total sleep time (5.6 ± 2.0 h) and high levels of fragmentation (sleep efficiency 68.4 ± 15.0%). Sleep interruption was reported on 71% of sleep diaries, with the most common reasons being due to medical care [measurement of vitals (23%), staff interruptions (22%), blood draws (21%)]. Those with lower sleep efficiency had more cognitive decline upon discharge. Although underpowered, there was a suggestion of an association with poor sleep and the likelihood of being discharged to a SNF. Those with worse self-reported sleep quality (PSQI) prior to admission had a slightly longer LOS. No associations were seen with sleep quality and likelihood of readmission.

**Conclusions:**

Collection of objective and subjective sleep measures was feasible among hospitalized older adults. Disrupted sleep was common, and was potentially related to poor hospital outcomes. Our next steps will be to leverage these results to design and implement an intervention to improve sleep in hospitalized adults.

## Introduction

Sleep disturbance in older adults is associated with increased risk of age-related health outcomes, including poorer cognition, increased risk of falls, and decreased wound repair (Blackwell et al., 2006, 2011, 2014; Stone et al., 2008, 2014; Diem et al., 2016; McLain et al., 2018; Cauley et al., 2019). Poor sleep during hospitalization is also related to increased risk of delirium (Weinhouse et al., 2009; Kamdar et al., 2016b), and is included as a risk factor in the Hospital Elder Life Program screening for delirium (Inouye et al., 2000). In a 2016 survey, 88% of critical care providers believed poor sleep could affect critically ill patients' recovery (Kamdar et al., 2016a). In a recent study, only 48% of section chiefs of hospital medicine who were interviewed about inpatient sleep responded that their facility had sleep friendly practices (Affini et al., 2022). In 2021, a multidisciplinary group of experts held an American Thoracic Society workshop to discuss research knowledge gaps, challenges and key next steps in causes and potential treatments for sleep and circadian disruption in the intensive care unit (Knauert et al., 2023). Although many reports have noted the need to improve sleep among inpatients (Stewart and Arora, 2018, 2021; Knauert et al., 2023), sleep in the hospital setting is understudied. There is limited literature to describe methods for measuring sleep under these circumstances (Hoey et al., 2014; Kamdar et al., 2017; Schwab et al., 2018).

Some prior studies have described sleep in hospital settings, including intensive care units (ICUs), surgical units, and acute care. Most have relied on subjective rating of sleep by the patient (Tranmer et al., 2003; Humphries, 2008; Yilmaz et al., 2012). Some have gathered objective data regarding sleep in small numbers of patients (Kroon and West, 2000; Shilo et al., 2000; Freedman et al., 2001; Tranmer et al., 2003; Taguchi et al., 2007; Yilmaz and Iskesen, 2007; Bourne et al., 2008; Mistraletti et al., 2009; Missildine et al., 2010; Chen et al., 2012; Yilmaz et al., 2012; Watson et al., 2013; Kamdar et al., 2017). Dres et al. (2019) measured sleep with polysomnography in 44 mechanically ventilated ICU patients. To our knowledge, no study has examined the association of objectively measured sleep during hospitalization with patient outcomes such as cognitive function, pain level, length of hospital stay (LOS), or the probability of readmissions (Schwab et al., 2018). If quality of sleep is associated with poor hospital outcomes, interventions should be designed to improve the hospital environment such that patients are able to obtain adequate sleep.

Factors associated with poor sleep in the hospital setting can be grouped as environmental, patient level (pain, anxiety, sleep history), and illness severity (Knauert et al., 2023). Many of these factors are potentially modifiable (Kamdar et al., 2014; Pisani et al., 2015). Minor changes to hospital workflow can improve environmental factors such as level of light exposure, minimizing noise, temperature level, and the amount of interaction with medical staff at night (Bano et al., 2014; Stewart and Arora, 2018).

We initiated a pilot study among older inpatients in an acute-care hospital that had no interventions in place related to sleep or circadian rhythms to describe sleep characteristics both objectively and subjectively. In addition, we used data from questionnaires and real-world evidence from electronic medical records and chart reviews to determine correlates and outcomes related to sleep disturbances. Results from this pilot study, as well as the lessons learned in its design and implementation, may be used to inform future interventions to improve sleep and quality of care for older adults in the hospital setting.

## Materials and methods

### Participants

The Sutter Tracy Community Hospital Sleep Study was a prospective study in an 81-bed acute-care hospital in Tracy, California with a goal of recruiting ~100 older adults from Medical/Surgical, Telemetry and Intensive Care Units. The study took place from January 2016 to November 2017. Recruitment was paused between February 2017 and June 2017 due to lack of study staffing. A dedicated staff research assistant was tasked with training and supervising student volunteers, recruited from nearby University of California, Davis. These student volunteers performed much of the participant recruitment and data collection.

Study staff examined patient census and attended grand rounds each morning to identify potentially eligible patients. Nursing staff introduced the study staff to these potential participants. Patients were eligible for the study if they were 60 years old or older. Exclusion criteria included: significant cognitive impairment, determined by screener, prior diagnosis, nurse decision or a score of <18 on the Montreal Cognitive Assessment (MoCA) (Nasreddine et al., 2005), a cut point established for detecting moderate to severe dementia, and selected because it may help capture early and late mild cognitive impairment stages (Trzepacz et al., 2015); inability to enroll the patient within 48 h of admission; language barrier (unable to understand English or Spanish); and an expected LOS that included less than two nights. Five months after study start, criteria were relaxed to allow enrollment of those with an LOS that included one night or more. There were no restrictions for specific hospital admission diagnoses, and patients were allowed to enroll in the study as many times as they were admitted to the hospital during the recruitment period. Study staff minimized risk of participant burden by allowing participants to take rest breaks as needed during the interview process, allowing participants to refuse to answer questions, and terminating the interview if the participant showed signs of distress or discomfort. Participants could withdraw from the study at any time. Study staff offered participants a $25 gift card mailed to their residence for compensation.

The Sutter Health Institutional Review Board approved the study. All participants provided written informed consent.

### Data collection

Study staff facilitated completion of questionnaire data at the time of enrollment, gathered subjective sleep diary data and information on pain level each day of the hospital stay, and administered tests of cognitive function at enrollment and discharge (data collection forms in Supplementary material). Study staff also applied wrist-worn actigraphs to collect objective sleep data on participants, gathered continuously from enrollment to discharge. Additional data were extracted from electronic health records and chart review where possible, to lessen participant burden.

### Subjective sleep measurement

Participants completed the Pittsburgh Sleep Quality Index (PSQI), a validated measure of subjective sleep quality and sleep disturbances covering the timeframe of 1 month prior to hospitalization. Global PSQI scores range from 0 to 21 and a score >5 is indicative of poor sleep (Buysse et al., 1989). The Epworth Sleepiness Scale (ESS), a self-administered questionnaire, was used to classify subjective daytime sleepiness for the participant's usual way of life in recent times prior to hospitalization. Scores on the ESS range from 0 to 24, with a score >10 indicating excessive daytime sleepiness (Johns, 1991, 1992).

Each day during the hospital stay following study enrollment, study staff collected minimal sleep diary data from participants, asking about when the participant tried to sleep at night and when they woke up in the morning, napping behavior, actigraph removals, and reasons for sleep interruptions during the previous night.

### Objective sleep measurement

Objective characteristics of sleep-wake patterns were estimated using an actigraph, which is similar in size to a wristwatch (Actiwatch^®^ 2 model, Philips Respironics, Inc., Murrysville, PA). Study staff placed the device on the non-dominant wrist of the participant, when possible. Participants wore the device continuously from enrollment until discharge. Prior studies have shown actigraphy provides a reliable estimate of sleep-wake patterns when compared to the gold-standard of polysomnography (Ancoli-Israel et al., 2003). In addition to measuring ambient light, the actigraph digitally recorded an integrated measure of gross motor activity using a solid-state piezoelectric accelerometer with a sensitivity of 0.025 G and a sampling rate of 23 Hz. The study protocol specified that activity measures were to be stored in 1-min epochs, however storage in 15 or 30 s epochs unintentionally occurred for some recordings. Actigraphy data that was collected in 15 or 30 s epochs [16 (19%)] were collapsed to 1-min epochs to ensure data from all participants was comparable. The sleep diary was used in the editing of the actigraphy data to determine when the participant tried to sleep at night and morning awakening, which were used to define the nighttime sleep interval, and when the actigraph was removed. Analysis of the actigraph data was conducted using the manufacturer's supplied software (Actiware, Philips Respironics, Inc., Murrysville, PA) (Actiware manual).^1^ A sleep scoring algorithm available in this software was used to determine sleep from wake times, calculating a moving average which accounted for the activity levels 2 min prior to and after the current minute to determine if each minute should be coded as sleep or wake (Oakley, 1997^2^; Kushida et al., 2001). Due to participant inactivity, the low threshold of 20 activity counts was used as the wake threshold. During the nighttime sleep interval, sleep onset was set to the first minute of a block of 20 min of continuous sleep and sleep offset (wake time) was set to the last minute scored as sleep.

Parameters estimated from actigraphy used in this analysis included sleep latency: minutes from the time the participant reported trying to sleep at night to sleep onset; total sleep time: the time per night spent sleeping during the nighttime sleep interval; sleep fragmentation measured with sleep efficiency (the percentage of time during the nighttime sleep interval spent sleeping) and wake after sleep onset (WASO, minutes of wake after sleep onset during the nighttime sleep interval). All parameters from actigraphy reflect data averaged over all nights they wore the device in order to obtain a more representative characterization of usual sleep patterns while hospitalized.

In addition to sleep parameters during the nighttime sleep interval, 24-h total sleep time was also estimated. The 24-h interval was defined as spanning 9:00 to 8:59 A.M. the following morning. Intervals with <20 h of valid data were not included in summaries. Data was averaged over all valid 24-h periods the participant wore the device.

### Outcomes measurement

Study staff used the MoCA to measure cognitive function, which evaluates memory, orientation, visuospatial functioning, concentration, calculation, attention, abstraction, language, and executive functions (Nasreddine et al., 2005). Scores range from 0 to 30, with higher scores representing better cognitive functioning. Change in MoCA score from enrollment to discharge was calculated. A negative change in MoCA score represents cognitive decline.

Collection of information on difficulty performing instrumental activities of daily living (IADL) and activities of daily living (ADL) at enrollment allowed for assessment of functional status. IADLs included walking two to three blocks on level ground, climbing up to ten steps, walking down 10 steps, preparing meals, doing heavy housework, and shopping for groceries or clothing. ADLs included dressing, getting in and out of beds or chairs, and bathing or showering (Pincus et al., 1983; Fitti and Kovar, 1987). Data on individual activities were collapsed to create dichotomous variables on presence or absence of any IADL, and similarly, presence or absence of any ADL.

Outcomes extracted from electronic health records included data on length of hospital stay (LOS), discharge to a skilled nursing facility (SNF), and re-admittance to a hospital within 30 days of discharge. To calculate LOS, the difference in days was calculated between admission and discharge dates such that each overnight stay counted as a 1 day LOS. Discharge disposition for each hospital encounter was used to identify individuals who were transferred to SNFs upon discharge. Lastly, readmission was identified as a binary variable representing inpatient admission to Sutter Tracy Community Hospital within 30 days of a participant's discharge. Additional outcomes extracted from chart review included patient falls during the hospital stay, and events of delirium, identified with the terms “delirium” and “encephalopathy”.

Participants self-reported pain level during the 24 h prior to enrollment, then for each day of the hospital stay. Participants used a horizontal visual analog scale, anchored by “no pain” with a score of zero to “worst possible pain” with a score of 100 (Hawker et al., 2011). Change in pain level from enrollment to the last pain measurement prior to discharge was calculated. A negative change represents a reduction in pain.

### Other measurements

All participants completed questionnaires at the time of enrollment, which included items about demographics, socio-economic status, self-reported prior clinical diagnosis of sleep disorders, and self-reported health status (data collection forms in Supplementary material). Depression was assessed using the number of depressive symptoms from the Geriatric Depression Scale (GDS), with higher scores corresponding to higher levels of depression and the standard cutoff of ≥6 symptoms used to define depression (Sheikh and Yesavage, 1986). The first measurement of body mass index (BMI) during the hospitalization was gathered from electronic health records. Obesity was defined as a BMI ≥ 30 kg/m^2^. International Classification of Diseases (ICD)-10 codes for primary diagnosis at admission were obtained from electronic health records.

### Statistical analysis

Participant characteristics, sleep parameters, and outcomes were summarized as means and standard deviations for continuous parameters, counts and percentages for categorical data.

The associations of the sleep parameters with the outcomes of discharge to a SNF, readmission within 30 days of discharge, and presence of an IADL or ADL at enrollment were assessed using logistic regression. Results are presented as odds ratios and 95% confidence intervals (OR, 95% CI). The associations of the sleep parameters with the outcomes of LOS, cognitive function at enrollment, change in cognitive function, pain level at enrollment and change in pain level were assessed using linear regression. Results are presented as beta coefficients and 95% CIs. All models were adjusted for age, race, sex and BMI. The significance of a quadratic term for 24-h total sleep time was explored in all models to examine if the potential associations were non-linear (U-shaped).

All significance levels reported were two-sided and all analyses were conducted using SAS version 9.4 (SAS Institute Inc., Cary, NC).

## Results

### Recruitment

**Figure 1:**
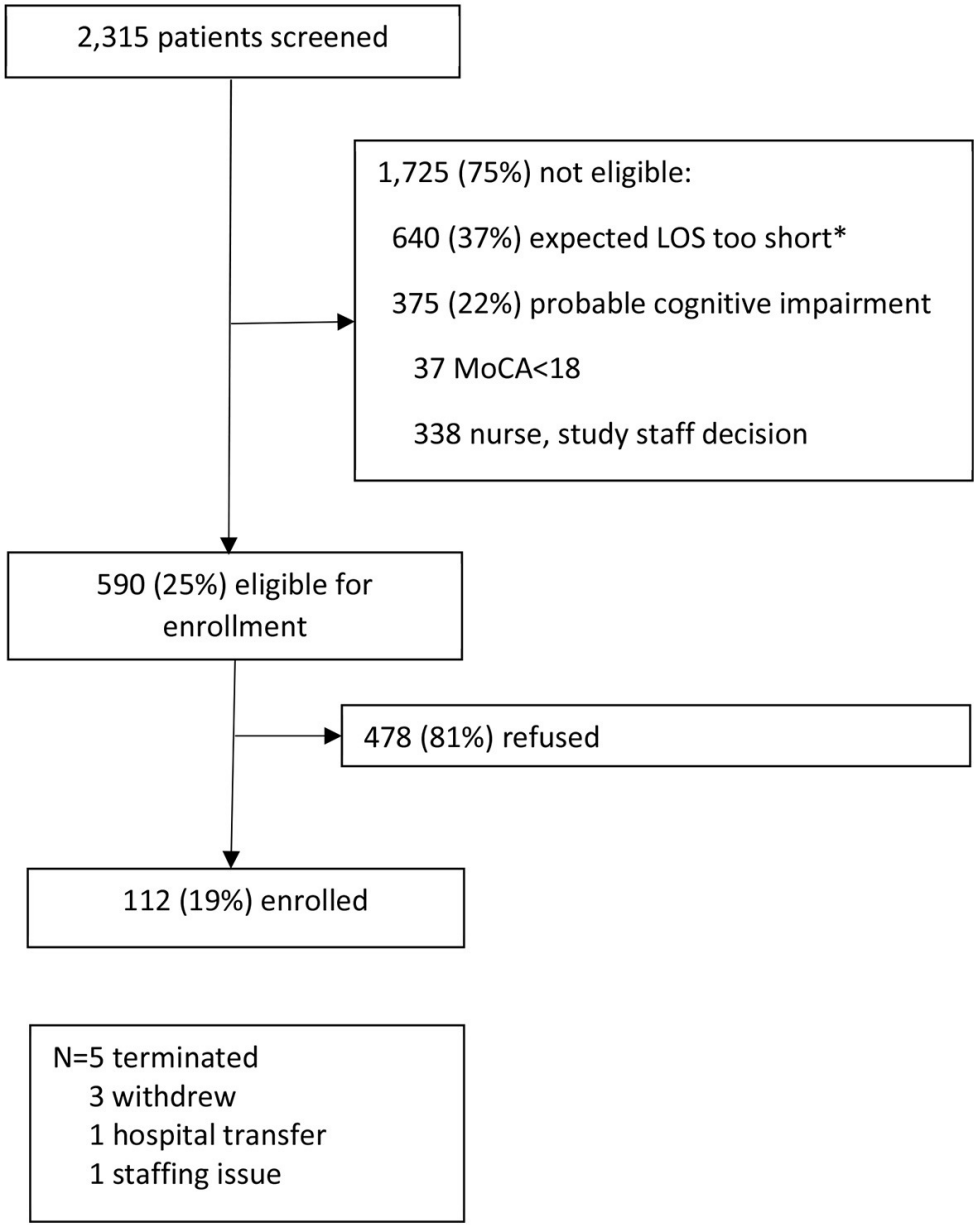
Enrollment process. *Exclusion criteria was initially expected length of hospital stay <2 days but was relaxed to <1 day after 5 months of recruitment. LOS, Length of hospital stay; MoCA, Montreal Cognitive Assessment.

Study staff screened 2,315 older patients for participation in the study over 19 months of recruitment (Figure 1). Of these, 75% were not eligible. The most common reason for ineligibility was that the expected LOS was not long enough (37% of ineligibles). Other reasons were probable cognitive impairment (22%) and language barrier (21%). Seven different languages were noted, with the most common being Spanish (*n* = 102). The explanations for those noted as “other” reason for ineligibility included that the patient was sleeping when recruitment was attempted (*n* = 41) and that the patient was in isolation (*n* = 36). Of those eligible for enrollment, 81% refused. Very little data was gathered on those who refused participation, and no reason for refusal was gathered. There were 112 patients (19% of those eligible) enrolled in the study. Of the 112 enrolled, five withdrew from the study. There were 104 unique patients in the study, with six patients participating in the study two times and one patient three times. Compared to those who participated in the study, those who refused had similar gender distribution (55 vs. 57% women, *p* = 0.067) but were older by an average of 5 years (*p* < 0.0001).

### Actigraphy data collection

Actigraphy was well-tolerated by the participants. Of the 112 participants, 111 were given the actigraph for objective sleep monitoring (Table 1). Most (89%) had actigraph recordings for all nights they were hospitalized. Eight-four (75%) of the participants had useable data. The reasons for failure to collect useable data were primarily device malfunction (*n* = 19, 68%). Of the 84 useable recordings, 68 (81%) were recorded in 1-min epochs as per protocol, 5 (6%) were gathered in 30-s epochs, and 11 (13%) in 15 s epochs. Data gathered in 15- or 30-s epochs were collapsed to 1-min epochs for comparability. Of those with data gathered on actigraph placement at enrollment, 40 (36%) participants had the device placed on the non-dominant wrist. Of those participants with the actigraph placed on the dominant wrist, 34 (56%) had the hospital identification band on the non-dominant wrist requiring placement of the actigraph on the dominant wrist.

**Table 1:** Actigraphy data collection, *n* (%).

**Parameter**	***N*(%)**
Wore actigraph	111 (99)
Placement on the non-dominant wrist	40 (36)
Had actigraphy for all nights	90 (89)
Had useable actigraphy data	84 (75)
**If not, why?**	
Accelerometer error^*^	17 (61)
Other device malfunction	2 (7)
Lost file	5 (18)
Not given actigraph	1 (4)
Patient removed actigraph each night	3 (11)
**Epoch length of recording**	
60 s	68 (81)
30 s	5 (6)
15 s	11 (13)

^*^Devices used were 9 years old or older.

### General descriptives

The average LOS for study participants was 3.4 ± 2.4 days (range 1–17), with most staying 3 or fewer days (66%) (Table 2). The most common diagnoses at admission were related to the cardiovascular (15.6%), respiratory (13.8%) or gastrointestinal systems (12.8%). The study participants were on average 69 years old (±7 years) with approximately equal proportions by gender. The majority of participants (77%) identified as white, with 12% reporting Hispanic origin. The average BMI was 31.6 ± 9.1 kg/m^2^, with half meeting criteria for obesity. Most lived in a private home or residence (94%), did not live alone (82%) and a little over half were married or in a married-like relationship (55%). Participants were asked to compare their socio-economic status to their peers in the community and the country, with an average score of 5.6 on a scale of 1–10, with 1 relating to people who are best off and 10 relating to people who are worse off. Regarding education, 18% of participants received a college degree of bachelor level or higher. Half rated their health status compared to others their age as fair or poor, and 29% met the criteria for depression. About a third of participants reported a prior clinical diagnosis of a sleep disorder (29%), with 25% reporting a prior diagnosis of sleep apnea.

**Table 2:** Patient characteristics, *n* (%) or mean ± SD.

**Characteristic**	**(*N* = 112)**
Age, years	68.7 ± 6.5
Of white race	75 (77.3)
Of Hispanic, Latino or Spanish origin	12 (12.1)
Sex	
Men	51 (45.5)
Women	61 (54.5)
Body mass index, kg/m^2^	31.6 ± 9.1
Body mass index ≥ 30 kg/m^2^	52 (50.5)
Participant lives alone	18 (18.0)
Type of residence participant lives in	
Private home or apartment	94 (94.0)
Retirement home or senior complex	4 (4.0)
Personal care home (assisted living)	1 (1.0)
Marital status	
Single, never married	8 (8.0)
Married or domestic partner	55 (55.0)
Widowed	15 (15.0)
Divorced	18 (18.0)
Separated	4 (4.0)
SES- where participant stands in the community (1–10)	5.6 ± 2.2
SES- where participant stands in USA (1–10)	5.6 ± 2.0
Education	
<High school diploma	12 (12.0)
High school graduate, GED	26 (26.0)
Some college or more	62 (62.0)
Self-reported health status	
Excellent/very good/good	50 (50.0)
Fair/poor	50 (50.0)
GDS score (range 0–15)	4.2 ± 3.2
Depression, GDS score ≥ 6	32 (29.0)
Self-report of clinical diagnosis of sleep disorder at admission	29 (29.3)
Restless legs syndrome	4 (4.0)
Sleep apnea	25 (25.3)
Insomnia	4 (4.0)
Periodic leg movement	3 (3.0)
Narcolepsy	0
Length of hospital stay, days	3.4 ± 2.4
Diagnosis at hospital admission^*^	
Cardiovascular	17 (15.6)
Chest pain	8 (7.3)
Shortness of breath	5 (4.6)
Syncope	2 (1.8)
Respiratory	15 (13.8)
Fever	3 (2.8)
Sepsis	4 (3.7)
Gastrointestinal	14 (12.8)
Orthopedic (osteoarthritis)	10 (9.2)
Other	31 (28.4)

SES, socio-economic status; GED, general equivalency diploma; GDS, Geriatric Depression Scale.

^*^Based on International Classification of Diseases (ICD)-10 code.

### Sleep characteristics

The majority of participants (77%) reported disturbed sleep the month prior to admission to the hospital (PSQI > 5) (Table 3). Just before admission participants reported getting 5.8 ± 2.0 h of sleep a night, on average. Patients self-reported taking an average of about half hour to fall asleep at night (33.3 ± 38.7 min) and spending only 72% of their time in bed at night sleeping (72.2 ± 23.3%). The majority of participants (69%) reported they did not use a sleep medication during the month prior to hospitalization. When asked about daytime sleepiness in recent times, 18% met the criteria for excessive daytime sleepiness (ESS > 10).

**Table 3:** Sleep characteristics^*^, *n* (%) or mean ± SD.

**Sleep parameter**	**(*N* = 112)**
**Pittsburgh Sleep Quality Index**	
Hours of actual sleep	5.8 ± 2.0
Sleep efficiency, %	72.2 ± 23.3
Sleep latency, min	33.3 ± 38.7
Sleep medication use	
Not during the past month	76 (68.5)
<1 time/week	3 (2.7)
1–2 times/week	6 (5.4)
3+ times/week	26 (23.4)
PSQI score (range 0–21)	9.1 ± 4.2
Poor sleep (PSQI > 5)	85 (76.6)
**Epworth Sleepiness Scale**	
ESS score (range 0–24)	6.7 ± 4.4
Excessive daytime sleepiness (ESS > 10)	20 (18.0)
**Participant takes naps regularly**	58 (52.3)
**Objective: Actigraphy**	(*N* = 84)
Number of nights of actigraphy data	2.2 ± 2.0
Total sleep time, hours	5.6 ± 1.9
Sleep efficiency, %	68.4 ± 15.0
Minutes of wake after sleep onset	87.7 ± 52.7
Sleep latency, min	85.6 ± 74.6
Number with 1+ 24-h period summary	58 (69.0)
Number of 24-h periods of data	2.0 ± 2.1
Total 24-h sleep time, hours	9.6 ± 3.1

PSQI, Pittsburgh Sleep Quality Index; ESS, Epworth Sleepiness Scale.

^*^PSQI was usual sleep habits the month prior to hospitalization; ESS was usual way of life in recent times prior to hospitalization. Objective sleep was measured during hospitalization.

Objective sleep measured during the hospitalization also showed disturbed sleep (Table 3).

On average, participants got 5.6 ± 1.9 h of sleep at night while hospitalized. Sleep was fragmented, with an average sleep efficiency of 68.4 ± 15.0%, and minutes of wake after sleep onset of 87.7 ± 52.7. Objectively measured time to fall asleep at night (sleep latency) was long (85.6 ± 74.6 min). The average 24-h total sleep time was 9.6 ± 3.1 h, so on average participants slept an additional 4 h of sleep outside the nighttime sleep interval.

Although the mean values were similar for subjectively measured sleep pertaining to the period just prior to admission and the objectively measured sleep during hospitalization, the correlation between the two was low (total sleep time *r* = 0.27, paired-test *p* = 0.11; sleep efficiency *r* = 0.06, paired-test *p* = 0.04; sleep latency *r* = 0.07, paired-test *p* < 0.01).

**Figure 2:**
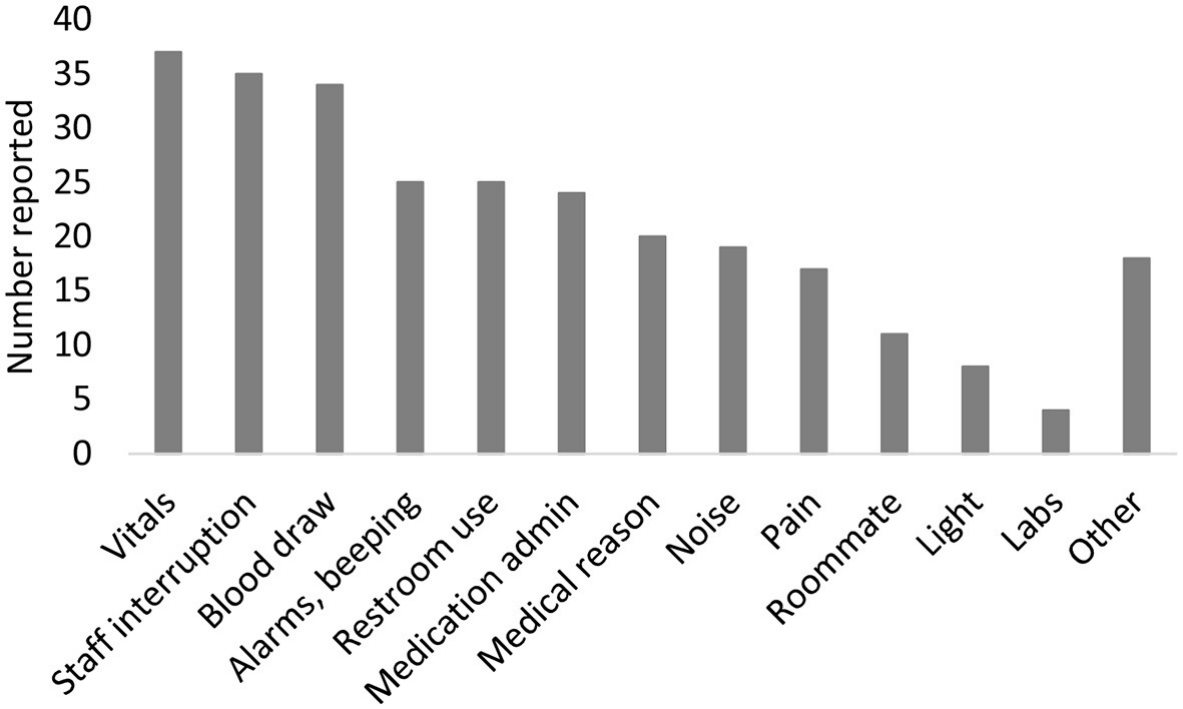
Reasons for sleep disruption. Participants can report >1 reason for sleep disruption each night.

Participants noted sleep interruptions during the prior night on the sleep diary (Figure 2). Of 226 diary entries, 160 (71%) reported sleep interruption. The most common reasons noted for sleep interruptions were due to medical care [measurement of vitals (23%), staff interruptions (22%), blood draws (21%), medication administration (15%), lab work (3%)] or trips to the bathroom (16%). Interruptions from environmental factors were also noted [noise (11%), machine noises (16%), light (5%)].

### Association of prevalent outcomes with self-reported sleep prior to admission

**Figure 3:**
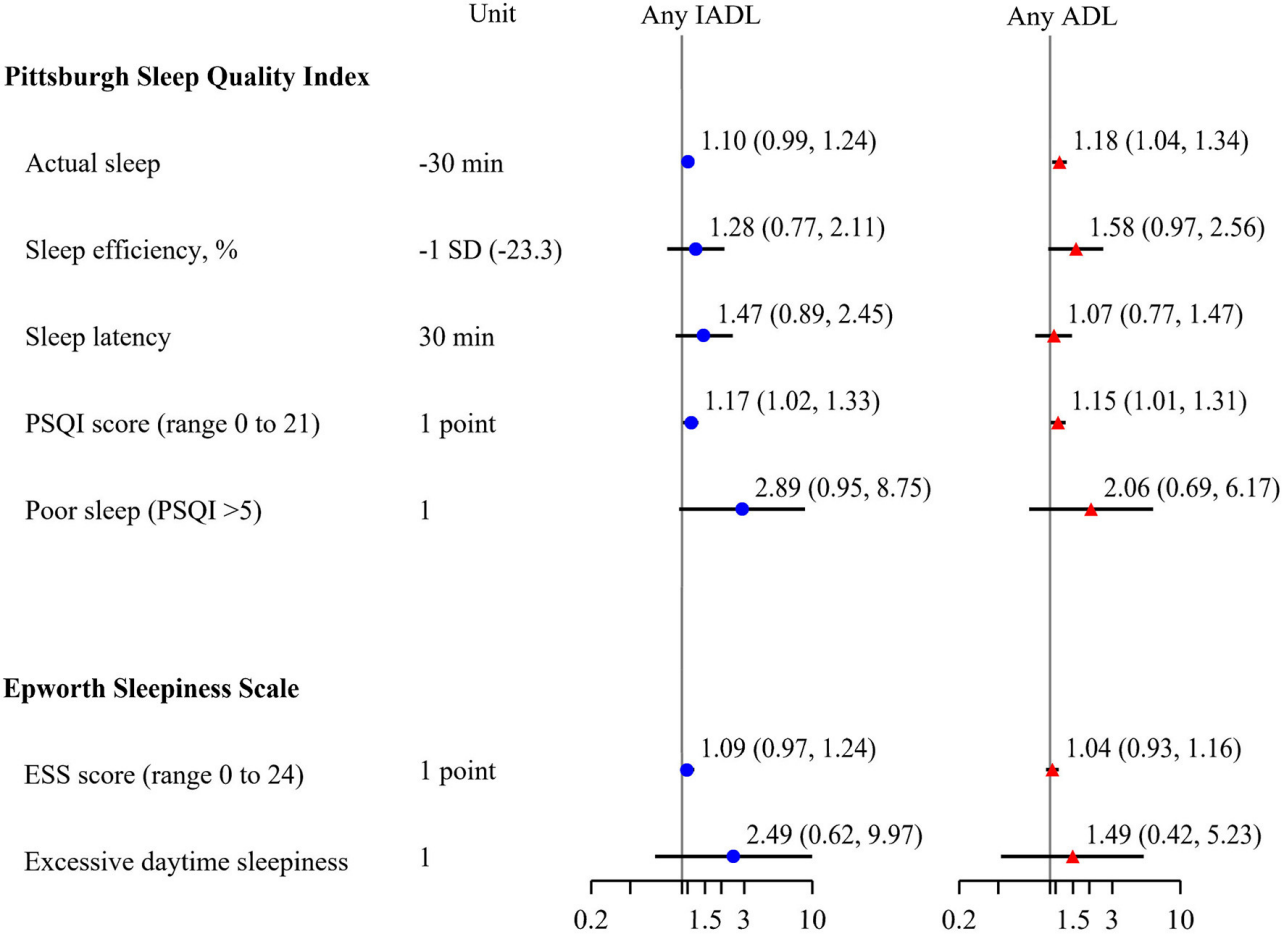
Multivariable adjusted associations of subjective sleep and presence of any IADLs or ADLs at admission, odds ratio (95% confidence interval). Models adjusted for age, race, sex, body mass index. PSQI, Pittsburgh Sleep Quality Index; ESS, Epworth Sleepiness Scale; IADL, Instrumental Activities of Daily Living; ADL, Activities of Daily Living.

The majority of participants had difficulty with one or more IADL (70%). The most common IADL difficulties were walking 2–3 blocks (55%), climbing 10 steps (56%) and doing heavy housework (56%). Close to half of participants also had difficulty with one or more ADL (48%), with 38% reporting difficulty getting into and out of bed. Self-reported sleep quality was associated with an increased risk of presence of one or more IADLs after multivariable adjustment (Figure 3). For each additional point on the PSQI the likelihood of having 1 or more IADL increased [OR (95% CI): 1.17 (1.02, 1.33)]. The association of poor sleep quality with ADLs was similar [OR (95% CI) per 1 point increase on PSQI: 1.15 (1.01, 1.31)]. Subjective total sleep time was associated with the likelihood of having an ADL, as measured by the PSQI [OR (95% CI) per 30 min decrease, 1.18 (1.04–1.34)]. There were no associations observed between daytime sleepiness and functional status.

On average, cognitive function was low at enrollment (23.4 ± 3.1). There were no associations observed between cognitive function level at enrollment and subjective sleep quality or daytime sleepiness (Table 4).

**Table 4:** Multivariable adjusted^*^ associations of subjective sleep and prevalent cognition or pain level.

		**Beta coefficient (95% confidence interval)**	
**Predictor**	**Unit**	**MoCA score (range to 30)**	**Pain score range (0 to 100)**
**Pittsburgh Sleep Quality Index**			
Actual sleep	−30 min	−0.001 (−0.16, 0.15)	0.59 (−1.21, 2.40)
Sleep efficiency, %	−1 SD (−23.3)	−0.41 (−1.04, 0.22)	6.48 (−0.86, 13.81)
Sleep latency	30 min	0.009 (−0.46, 0.48)	5.89 (0.45, 11.33)^**^
PSQI score (range 0–21)	1 point	−0.08 (−0.24, 0.08)	2.42 (0.65, 4.21)^***^
Poor sleep (PSQI > 5)	1	−0.29 (−1.77, 1.19)	21.71 (5.00, 38.41)^**^
**Epworth Sleepiness Scale**			
ESS score (range 0–24)	1 point	−0.04 (−0.18, 0.10)	−0.08 (−1.77, 1.61)
Excessive daytime sleepiness (ESS > 10)	1	0.12 (−1.57, 1.82)	−3.55 (−24.23, 17.13)

^*^Adjusted for age, race, sex, body mass index.

^**^p < 0.05, ^***^p < 0.01.

MoCA, Montreal Cognitive Assessment; SD, standard deviation; PSQI, Pittsburgh Sleep Quality Index; ESS, Epworth Sleepiness Scale.

PSQI was usual sleep habits the month prior to hospitalization; ESS was usual way of life in recent times prior to hospitalization.

On average, the pain level reported at enrollment was 52.4 ± 33.8 on a scale of 0–100, with 31.5% considered to be in severe pain (≥75) (Hawker et al., 2011). After adjustment for confounders, for each additional point increase on the PSQI the pain level was greater by 2.42 (*p* < 0.01) (Table 4). Those meeting the criteria for poor self-reported sleep reported higher pain levels by an average of 21.7 (*p* < 0.05). Longer self-reported sleep latency was related to higher pain levels at enrollment. There were no associations observed between daytime sleepiness and pain level at enrollment.

### Association of incident outcomes happening after admission with subjective and objective sleep

**Figure 4:**
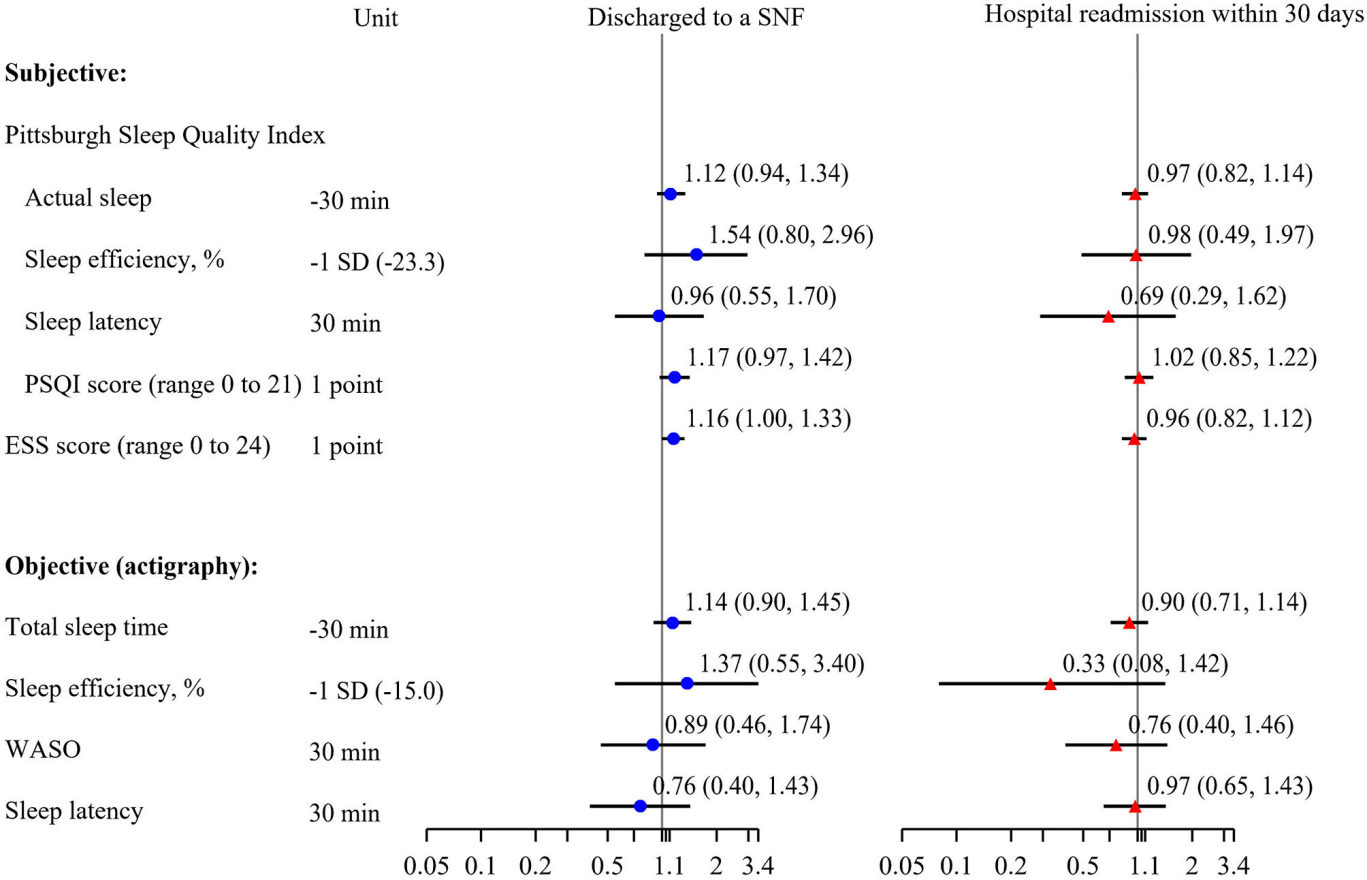
Multivariable adjusted associations of subjective sleep and objective sleep and incidence of discharge to a skilled nursing facility or readmission to the hospital within 30 days of discharge, odds ratio (95% confidence interval). Models adjusted for age, race, sex, body mass index. PSQI, Pittsburgh Sleep Quality Index; ESS, Epworth Sleepiness Scale; WASO, wake after sleep onset; SNF, skilled nursing facility.

Although our pilot study was not powered to identify statistically significant associations between sleep and incident outcomes, we explored these associations to determine any trends that could inform future studies. Of those with follow-up data from electronic health records (*n* = 108), 11 (10.2%) were discharged to a SNF and 14 (13.0%) were readmitted to a hospital within 30 days of discharge. The small number of events make adjustment for potential confounders difficult. Adjusted models with objectively measured sleep parameters had *n* = 68 participants with *n* = 7 participants that were discharged to a SNF and *n* = 7 readmissions, while adjusted models with subjective sleep parameters had *n* = 88 participants with *n* = 10 discharged to a SNF and *n* = 10 readmissions. Though not statistically significant, those with lower levels of objectively measured total sleep time and sleep efficiency had higher odds of SNF placement (Figure 4). For each point increase on both the subjective disturbed sleep and daytime sleepiness scales, there was a 16–17% increase in likelihood to be discharged to a SNF [OR (95% CI) for: PSQI 1.17 (0.97, 1.42) *p* = 0.11; ESS 1.16 (1.00, 1.33) *p* = 0.04]. Associations of these sleep parameters with likelihood of readmissions were not statistically significant and had effect sized suggesting a null relationship. For each point increase on the subjective sleep quality scale (PSQI) there was an increase in LOS by 0.13 days on average (*p* < 0.05). Associations of 24-h total sleep time with the likelihood of SNF placement or readmission were not significant (data not shown).

Cognitive testing was to be performed a second time the day of discharge. Seventy-four (66%) participants completed this cognitive testing. Of those missing these data, four were not expected to complete the test because they withdrew or were transferred to another hospital, 18 were discharged before the test could be completed, six refused to be tested and 10 have an unknown reason for missing data. Overall, cognition did improve by time of discharge. On average, the MoCA scores increased by 1.3 ± 2.9 points. There were no associations between subjective sleep prior to admission and change in cognitive function during hospitalization. After adjustment for confounders, each standard deviation decrease in objectively measured sleep efficiency was associated with a decline of about 1 point on the MoCA, and each 30-min decrease in 24-h total sleep time was related to a 0.16 point decline on the MoCA (*p* < 0.05) (Table 5).

**Table 5:** Multivariable adjusted^*^ associations of subjective and objective sleep and incident length of stay, change in cognition and change in level of pain.

		**Beta coefficient (95% confidence interval)**		
**Predictor**	**Unit**	**Length of hospital stay**	**Change in cognitive function**	**Change in pain score**
**Subjective: Pittsburgh Sleep Quality Index**				
Actual sleep	−30 min	0.09 (−0.05, 0.20)	−0.11 (−0.29, 0.08)	0.09 (−1.69, 1.86)
Sleep efficiency, %	−1 SD (−23.3)	0.31 (−0.21, 0.82)	−0.60 (−1.34, 0.15)	0.54 (−6.74, 7.83)
Sleep latency	30 min	−0.08 (−0.46, 0.30)	−0.11 (−0.82, 0.60)	2.32 (−3.05, 7.69)
PSQI score (range 0–21)	1 point	0.13 (0.002, 0.25)^**^	−0.13 (−0.30, 0.05)	0.90 (−0.90, 2.70)
**Subjective: ESS**				
ESS score (range 0–24)	1 point	−0.06 (−0.18, 0.05)	−0.11 (−0.27, 0.05)	0.37 (−1.27, 2.02)
**Objective: Actigraphy**				
Total sleep time	−30 min	−0.21 (−0.64, 0.23)	−0.13 (−0.34, 0.09)	0.12 (−2.07, 2.32)
Sleep efficiency, %	−1 SD (−15.0)	−0.09 (−0.68, 0.50)	−0.95 (−1.73, −0.17)^**^	−0.33 (−9.16, 8.51)
WASO	30 min	−0.02 (−0.42, 0.38)	−0.54 (−1.08, −0.003)	−2.55 (−8.56, 3.45)
Sleep latency	30 min	−0.14 (−0.39, 0.11)	−0.20 (−0.51, 0.12)	1.15 (−3.49, 5.80)
24–h total sleep time	−30 min	0.002 (−0.12, 0.12)	−0.16 (−0.31, −0.02)^**^	0.11 (−1.56, 1.77)

^*^Adjusted for age, race, sex, body mass index.

^**^p < 0.05.

SD, Standard deviation; PSQI, Pittsburgh Sleep Quality Index; ESS, Epworth Sleepiness Scale; WASO, wake after sleep onset.

PSQI was usual sleep habits the month prior to hospitalization; ESS was usual way of life in recent times; Objective sleep was measured during hospitalization.

Pain level was considerably reduced by the last day of the hospital stay, with an average reduction of 13.9 points. The rate of those in severe pain was reduced by half. There were no associations of subjective sleep prior to admission or objectively measures sleep during hospitalization and change in pain level (Table 5).

All quadratic terms examined for 24-h total sleep time were not significant.

There were few instances of delirium (*n* = 2) or falls (*n* = 1) while hospitalized, preventing any statistical inference.

## Discussion

Our study found that, on average participants in this sample of older hospitalized patients had disturbed sleep, in both self-reported sleep quality just prior to hospitalization and objectively measured sleep quality during hospitalization. Those with lower self-reported sleep quality just before admission had a higher likelihood of having one or more IADLs or ADLs and a higher level of pain. Although underpowered, there is a suggestion of an association between sleep disturbance and greater likelihood of discharge to a SNF. There were no apparent associations with sleep and the risk of readmission within 30 days. However, there was an association between poor subjective sleep quality before admission and longer LOS. This study adds to current knowledge by exploring associations of objectively measured sleep in the hospital setting with patient outcomes (Schwab et al., 2018).

Participants in this sample of older hospitalized patients had disturbed sleep, compared to sleep characteristics from cohorts of community dwelling men and women of similar age or older (Blackwell et al., 2006, 2011; Beaudreau et al., 2012). Women in the Study of Osteoporotic Fractures (SOF) (average age 83 years, 89% white) reported an average PSQI of 6.3 ± 3.6 and a median ESS of 5. Men in the Outcomes of Sleep Disorders in Older Men (MrOS Sleep) Study (average age 77 years, 90% white) reported an average PSQI of 5.6 ± 3.3 and an average ESS of 6.2 ± 3.7. In comparison, the patients in the current study reported much worse sleep just prior to hospitalization (PSQI 9.1 ±4.2, ESS 6.7 ± 4.4). Objectively measured sleep during hospitalization was also qualitatively much worse, on average, than sleep measured among these community dwelling cohorts. Total sleep time was shorter by about an hour (Current study, SOF, MrOS: 5.6 ± 1.9 h, 6.7 ± 1.3 h, 6.4 ± 1.2 h). Sleep was also more fragmented (WASO, min. Current study, SOF, MrOS: 87.7 ± 52.7, 77.2 ± 48.0, 78.4 ± 44.3).

There was a significant reduction in pain by the end of the hospitalization. Self-reported sleep measures were associated to pain level at enrollment, but neither self-reported nor objectively measured sleep were associated to change in pain level from enrollment to discharge. While the association of sleep and pain is considered to be bi-directional, some studies suggest sleep is a stronger predictor of pain than vice versa (Finan et al., 2013).

There was no association observed between cognition and subjective sleep. Overall, cognition improved from admission to discharge by an average of 1.3 points on the MoCA, which can be considered a clinically meaningful difference (Krishnan et al., 2017). There was an association with higher levels of objectively measured sleep fragmentation and decline in cognition, as was found in other studies (Blackwell et al., 2014; Diem et al., 2016). Decline in cognition in those with fragmented sleep during hospitalization potentially highlights the importance of improving sleep to restore cognitive function by time of discharge. Both sleep deprivation and cognitive impairment are considered risk factors for development of delirium in the hospital setting, stressing the importance of interventions for their improvement (Inouye et al., 2000). Longer-term studies are needed to determine the duration of cognitive impairment after hospitalization in relation to sleep disturbance. It is possible the overall improvement in cognition during hospitalization was due to a learning effect because of the relatively short interval between exams. Future studies examining sleep in the hospital setting and cognitive function and decline should include a more complete battery of tests, that are robust to a learning effect.

A goal of the current study was to examine the feasibility of collecting objective sleep data and questionnaire data in the hospital setting, to be used in combination with real-world evidence from electronic health record data. We successfully met our recruitment and data collection goals and found objective sleep assessment was possible, as was also found in another feasibility study (Kamdar et al., 2017). Strong relationships between study and hospital staff were the foundation to successful recruitment. We would, however, consider doing several things differently in future studies. We would include more reasons for non-enrollment on our data collection forms, gather information on reasons for refusal to participate and attempt to gather basic demographic information on those who refused participation. We were unable to obtain cognitive assessment at discharge for approximately one third of our participants, with the most common reason being that the participant had been discharged before data could be collected. Perhaps collecting follow-up data at more frequent intervals would have allowed for the collection of longitudinal data on more participants.

Utilization of actigraphy for collection of objective sleep data can be successfully implemented in the hospital setting. Due to budget restrictions, we did not purchase new actigraphs for this study. Using older equipment led to the loss of data due to equipment failure. Due to the inactivity of the patients, use of actigraphs with the most sensitive accelerometers would be preferable. Our protocols did not specify to attempt to leave the light meter on the actigraph uncovered, which would have allowed for better use of this data. Our protocol stated it was preferable to place the actigraph on the non-dominant wrist, but we found that this was not always possible because of placement of hospital identification bands and vascular access, among other reasons. Other studies using actigraphy in the hospital setting have had similar issues with actigraph placement (Missildine et al., 2010; Kamdar et al., 2017). Some studies have found there was no difference in sleep data collected by actigraphs placed on the dominant and non-dominant wrist (Ancoli-Israel et al., 2003). While there were no complaints about discomfort from the actigraph, we would like to have formally asked this, as well as information about participant burden. We gathered subjective sleep quality data regarding the period just prior to hospitalization, but did not gather it again during the hospital stay or at discharge making comparisons impossible. Also, information regarding sleep quality and quantity after discharge would be informative.

There were some limitations to this pilot study. While larger than most prior studies of sleep in the hospital setting, this current study was underpowered to examine associations of sleep quality with dichotomous outcomes. The study took place in one hospital, so results may not be generalizable to hospitals in other areas. It is possible the overall improvement in cognition during hospitalization was due to a learning effect because of the relatively short interval between exams. We did not gather information on medication use, specifically sleep medications, benzodiazepine use and opioid use, which effect the amount and quality of sleep. The definition of delirium was not systematically collected or reliably assessed from electronic health record data. The addition of a validated delirium assessment tool such as the Confusion Assessment Method (CAM) would have been an improvement to our study (Lin et al., 2023). Objectively measured sleep latency relies on accuracy of the participant reporting the time they tried to sleep on the sleep diaries, which may have been difficult for the participant to recall. Polysomnography is more accurate for assessing sleep than actigraphy, however, polysomnography has been considered infeasible to use in the hospital setting due to the equipment needs, cost, and patient burden (Kamdar et al., 2017). The sleep quality questionnaire used in our study has a number of questions and takes 5–10 min (Buysse et al., 1989). We could have used the Richards-Campbell Sleep Questionnaire, to be gathered daily, which takes about 2 min to complete (Richards et al., 2000). It would have been easier to quantify reason for sleep disruption if we had preset categories, rather than having to glean it from text. We also could have asked the nurses their opinion on the sleep quality of the patient. Some participants (25%) reported a diagnosis of sleep apnea, which has been shown to be related to both sleep fragmentation and cognitive disfunction (Yaffe et al., 2014; Seda and Han, 2020). In order to disentangle the effect of sleep fragmentation on outcomes, future studies may exclude those with clinically significant sleep apnea, or gather more complete information regarding the severity and treatment of sleep apnea at admission. It would be informative to study sleep in the hospital setting more effectively by implementing changes to our protocol to address these limitations.

In conclusion, the completion of this pilot study has demonstrated that collection of objective and subjective measures of sleep is feasible among hospitalized older adults. We were able to measure sleep quantitatively on more than 100 patients. Poor sleep, including inadequate nighttime sleep duration and more fragmented sleep, was common in hospitalized older adults. Based on patient reports, the most common reasons for disrupted sleep were alarms/beeping, staff interruptions (e.g., blood draws, vitals measurements), pain or other medical reasons, restroom use, and medication administration. Potential future directions may include interventions targeted at patient care changes for hospital staff, for example, limiting sleep interruptions by reducing nighttime care and avoiding overhydration to reduce patient nighttime restroom use. Other intervention components may include automating the blood draw process with a mechanism that allows non-invasive, multiple blood draws with a single needle stick. Environmental interventions could include guidelines for light exposure and noise reduction. By intervening, it may be possible to improve sleep and health quality during hospitalization, but also outcomes such as cognition and shorter LOS.

## Data availability statement

The datasets presented in this article are not readily available because they contain patient health information that could compromise the privacy of research participants. Requests to access the datasets should be directed to tblackwell@sfcc-cpmc.net.

## Ethics statement

The studies involving humans were approved by Sutter Health Institutional Review Board. The studies were conducted in accordance with the local legislation and institutional requirements. The participants provided their written informed consent to participate in this study.

## Author contributions

TB: Conceptualization, Data curation, Formal analysis, Writing – original draft, Writing – review & editing. SR: Conceptualization, Data curation, Formal analysis, Writing – original draft, Writing – review & editing. NT: Data curation, Supervision, Writing – original draft, Writing – review & editing. LD-G: Conceptualization, Project administration, Writing – original draft, Writing – review & editing. PY: Conceptualization, Funding acquisition, Writing – original draft, Writing – review & editing. AP: Conceptualization, Writing – original draft, Writing – review & editing. KS: Conceptualization, Writing – original draft, Writing – review & editing.

## Footnotes

^1^Actiware and Actiware CT Software Manual, Respironics, Inc. Bend, OR.

^2^Oakley, N. R. (1997). *Validation With Polysomnography of the Sleepwatch Sleep/Wake Scoring Algorithm Used by the Actiwatch Activity Monitoring System*. Technical rePort to Mini Mitter Co., Inc.
