# Supplementary material for: Objective and subjective sleep characteristics in hospitalized older adults and their associations to hospital outcomes
Source: Front Sleep. 2024 Apr 5;3:1346642. doi: 10.3389/frsle.2024.1346642 (PMC12713864; doi:10.3389/frsle.2024.1346642)
Supplement: Supplementary file 1 [file Data_Sheet_1.PDF]

Supplementary Material, Data Collection Forms

Objective and subjective sleep characteristics in hospitalized older adults and their associations to hospital outcomes

Terri L. Blackwell[1]*, Sarah Robinson[2], Nicholas Thompson[3], Lisa Dean-Gilley[2],Phillip Yu[3], Alice Pressman[2], Katie L. Stone[1]

Affiliations:

[1]Sutter Health California Pacific Medical Center Research Institute, San Francisco, CA.

[2]Sutter Health, Center for Health Systems Research, Walnut Creek, CA.

[3]Sutter Tracy Community Hospital, Tracy, CA.

Patient Intake Form

Record ID:____________

Patient Identification
Patient MRN:______________
Patient Name:__________________________
Date of Birth: _______/________/__________
Age:__________
If the patient was not enrolled in the study, please identify why:

- □ Patient refused to participate
- □ Patient was not enrolled within the 48 hour time period
- □ Patient did not pass the MoCA
- □ Other: ____

Study Events
Date of Admission: _______/________/__________
Date of Enrollment: _______/________/__________
Date of Closeout: _______/________/__________
Reason for Closeout:

- ○ Patient withdrew from the study
- ○ Patient was discharged
- ○ Patient was enrolled for the max number of days (14 days)
- ○ Other : ____

Date of Discharge: _______/________/__________

Admission Form Packet

These standardized and validated tests/questionnaires were completed at part of the Admission process. Please see the references for information on the forms:

Montreal Cognitive Assessment (MoCA)

Reference: Nasreddine, Z. S., Phillips, N. A., Bédirian, V., Charbonneau, S., Whitehead, V., Collin, I., et al. (2005). The Montreal Cognitive Assessment, MoCA: a brief screening tool for mild cognitive impairment. *Journal of the American Geriatrics Society*, *53*(4), 695–699. https://doi.org/10.1111/j.1532-5415.2005.53221.x

The Pittsburgh Sleep Quality Index (PSQI)

Buysse, D. J., Reynolds, C. F., 3rd, Monk, T. H., Berman, S. R., and Kupfer, D. J. (1989). The Pittsburgh Sleep Quality Index: a new instrument for psychiatric practice and research. *Psychiatry research*, *28*(2), 193–213. https://doi.org/10.1016/0165-1781(89)90047-4

The Epworth Sleepiness Scale (ESS)

Johns M. W. (1991). A new method for measuring daytime sleepiness: the Epworth sleepiness scale. *Sleep*, *14*(6), 540–545. https://doi.org/10.1093/sleep/14.6.540

Johns M. W. (1992). Reliability and factor analysis of the Epworth Sleepiness Scale. *Sleep*, *15*(4), 376–381. https://doi.org/10.1093/sleep/15.4.376

The Geriatric Depression Scale (GDS)

Sheikh J, and Yesavage J. Geriatric Depression Scale: recent evidence and development of a shorter version. Clinical Gerontology: A Guide to Assessment and Intervention. New York: The Haworth Press, 1986. pp 165-173.

Think of this ladder as representing where people stand in the United States.

At the **top** of the ladder are the people who are the best off - those who have the most money, the most education and the most respected jobs. At the **bottom** are the people who are the worst off - who have the least money, least education, and the least respected jobs or no job. The higher up you are on this ladder, the closer you are to the people at the very top; the lower you are, the closer you are to the people at the very bottom.

Where would you place yourself on this ladder?

Please place a large "**X**" on the rung where you think you stand at this time in your life, relative to other people in the United States.

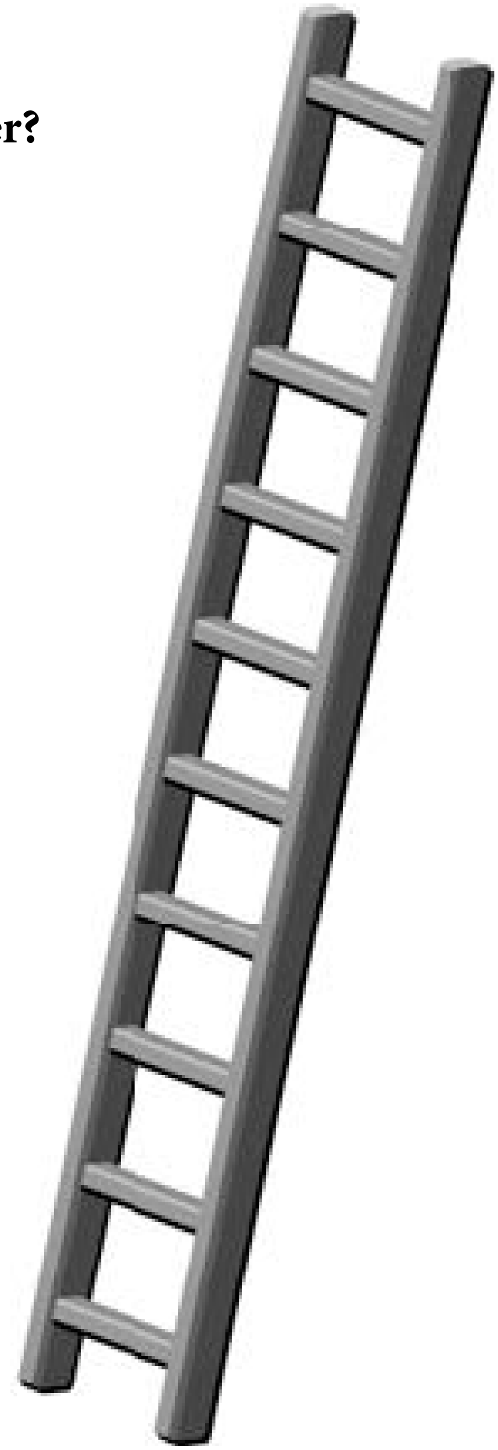

Think of this ladder as representing where people stand in their communities.

People define community in different ways; please define it in whatever way is most meaningful to you. At the **top** of the ladder are the people who have the highest standing in their community. At the **bottom** are the people who have the lowest standing in their community.

Where would you place yourself on this ladder?

Please place a large "**X**" on the rung where you think you stand at this time in your life, relative to other people in your community.

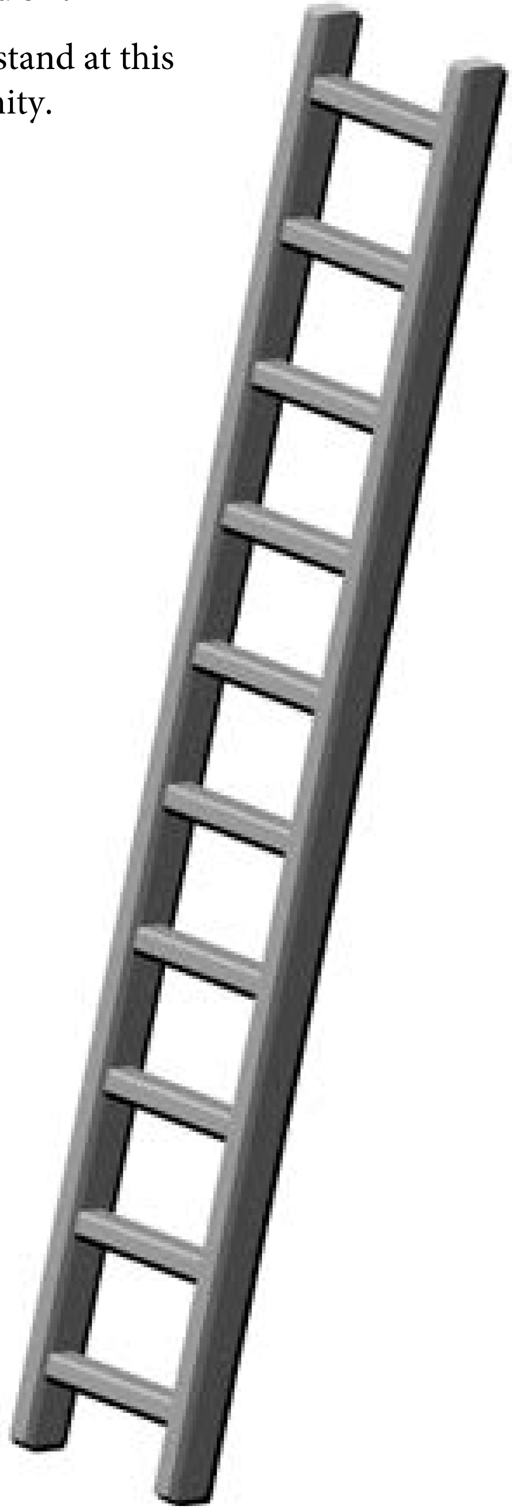

Sleep Habits

The folllowing questions refer to your usual sleep patterns prior to hospitalization.

1. **On most nights, how many hours do you sleep each night?**

   ☐☐ **hours**

2. **How many hours of sleep do you <u>need</u> each night to feel rested? (Please answer to the nearest hour.)**

   ☐☐ **hours**

3. **Do you take naps regularly?**

   ○ Yes ○ No ○ Don't know

   a. How many days per week do you usually nap? ☐ **days**

   b. On average, how many hours do you nap each time?

   ○ Less than 1 hour ○ At least 1 hour but no more than 2 hours ○ More than 2 hours

4. **Do you ever drink alcohol to help you sleep?** ○ Yes ○ No ○ Don't know

Actigraph Placement

Are you left handed or right handed?

- □ Right handed
- □ Left handed

The following questions should be completed by the research staff:

Which side was the actigraph placed on?

- □ Right
- □ Left

Which side is the identification band on?

- □ Right
- □ Left

Functional Status

These questions are about how well you are able to do certain activities <u>by yourself and without the use of aids</u>.

1 Do you have <u>ANY</u> difficulty walking 2 or 3 blocks outside on level ground?

○ Yes ○ No ○ I don't do it

GO TO QUESTION #2

a. Is this because of a health or physical problem? ○ Yes ○ No ○ Don't know

b. By yourself, and without using aids, how much difficulty do you have doing this?

○ Some difficulty ○ Much difficulty ○ Unable to do it ○ Don't know

c. Do you receive help from another person when you do this? ○ Yes ○ No ○ Unable/doesn't do it

2 Do you have <u>ANY</u> difficulty climbing up 10 steps without resting?

○ Yes ○ No ○ I don't do it

GO TO QUESTION #3

a. Is this because of a health or physical problem? ○ Yes ○ No ○ Don't know

b. By yourself, and without using aids, how much difficulty do you have doing this?

○ Some difficulty ○ Much difficulty ○ Unable to do it ○ Don't know

c. Do you receive help from another person when you do this? ○ Yes ○ No ○ Unable/doesn't do it

3 Do you have <u>ANY</u> difficulty walking down 10 steps?

○ Yes ○ No ○ I don't do it

GO TO QUESTION #4

a. Is this because of a health or physical problem? ○ Yes ○ No ○ Don't know

b. By yourself, and without using aids, how much difficulty do you have doing this?

○ Some difficulty ○ Much difficulty ○ Unable to do it ○ Don't know

c. Do you receive help from another person when you do this? ○ Yes ○ No ○ Unable/doesn't do it

Functional Status

4 **Do you have ANY difficulty preparing your own meals?**

○ Yes ○ No ○ I don't do it

GO TO QUESTION #5 (if No)

a. Is this because of a health or physical problem? ○ Yes ○ No ○ Don't know

b. By yourself, and without using aids, how much difficulty do you have doing this?

○ Some difficulty ○ Much difficulty ○ Unable to do it ○ Don't know

c. Do you receive help from another person when you do this? ○ Yes ○ No ○ Unable/doesn't do it

5 **Do you have ANY difficulty doing heavy housework (like scrubbing the floors or washing windows)?**

○ Yes ○ No ○ I don't do it

GO TO QUESTION #6 (if No)

a. Is this because of a health or physical problem? ○ Yes ○ No ○ Don't know

b. By yourself, and without using aids, how much difficulty do you have doing this?

○ Some difficulty ○ Much difficulty ○ Unable to do it ○ Don't know

c. Do you receive help from another person when you do this? ○ Yes ○ No ○ Unable/doesn't do it

6 **Do you have ANY difficulty doing your own shopping for groceries and clothes?**

○ Yes ○ No ○ I don't do it

GO TO QUESTION #7 (if No)

a. Is this because of a health or physical problem? ○ Yes ○ No ○ Don't know

b. By yourself, and without using aids, how much difficulty do you have doing this?

○ Some difficulty ○ Much difficulty ○ Unable to do it ○ Don't know

c. Do you receive help from another person when you do this? ○ Yes ○ No ○ Unable/doesn't do it

Functional Status

7 Do you have ANY difficulty getting in or out of bed or chairs?

○ Yes ○ No ○ I don't do it

GO TO QUESTION #8

a. Is this because of a health or physical problem? ○ Yes ○ No ○ Don't know

b. By yourself, and without using aids, how much difficulty do you have doing this?

○ Some difficulty ○ Much difficulty ○ Unable to do it ○ Don't know

c. Do you receive help from another person when you do this? ○ Yes ○ No ○ Unable/doesn't do it

8 Do you have ANY difficulty dressing?

○ Yes ○ No ○ I don't do it

GO TO QUESTION #9

a. Is this because of a health or physical problem? ○ Yes ○ No ○ Don't know

b. By yourself, and without using aids, how much difficulty do you have doing this?

○ Some difficulty ○ Much difficulty ○ Unable to do it ○ Don't know

c. Do you receive help from another person when you do this? ○ Yes ○ No ○ Unable/doesn't do it

9 Do you have ANY difficulty bathing or showering?

○ Yes ○ No ○ I don't do it

GO TO QUESTION #10

a. Is this because of a health or physical problem? ○ Yes ○ No ○ Don't know

b. By yourself, and without using aids, how much difficulty do you have doing this?

○ Some difficulty ○ Much difficulty ○ Unable to do it ○ Don't know

c. Do you receive help from another person when you do this? ○ Yes ○ No ○ Unable/doesn't do it

Functional Status

(10) **Ask the participant if they use any of the following aids when doing any of the activites listed above. If yes, amend part b of questions that apply.**

**Cane**	**Jar opener for previously opened jars**
**Walker**	**Special eating utensils**
**Crutches**	**Long-handled appliance for reach or in the bathroom**
**Wheelchair**	**Bathtub seat or bar**
**Special or built up chair**	**Raised toilet seat**
**Devices for dressing like button hooks, zipper pulls, etc.**	

(11) **During the past 12 months, on a typical night, how many times do you get up to go to the bathroom to empty your bladder (from the time you go to sleep until you wake up in the morning)?**

- ○ 0
- ○ 1-2
- ○ 3-4
- ○ 5 or more times

Admission Questionnaire

1) What is your race or origin? (Please select all that apply)
- □ White
- □ Black/African American
- □ American Indian or Alaskan Native
- □ Asian
- □ Native Hawaiian or Other Pacific Islander
- □ Some other Race
- □ Decline to Answer

2) Are you of Hispanic, Latino, or Spanish origin?
- ○ No
- ○ Yes
- ○ Decline to Answer

3) What is your marital status?
- ○ Single, never married
- ○ Married or domestic partnership
- ○ Widowed
- ○ Divorced
- ○ Separated
- ○ Decline to Answer

4) What is the highest degree or level of school you have completed?
- ○ Less than high school diploma
- ○ High school graduate, GED, or alternative
- ○ Some college or associate's degree
- ○ Bachelor's degree
- ○ Some graduate school (e.g. Master’s degree, Professional school degree, Doctoral degree)
- ○ Graduate school degree (e.g. Master’s degree, Professional school degree, Doctoral degree)
- ○ Decline to Answer

5) What was your family income in the past 12 months:
- ○ Less than $10,000
- ○ $10,000 to $24,999
- ○ $25,000 to $49,999
- ○ $50,000 to $99,999
- ○ $100,000 to $149,999
- ○ $150,000 or more
- ○ Decline to Answer

6) Which of the following statements about occupational status apply to you?
- ○ Not working at the moment
- ○ Part-time or hourly work < 15 hours per week
- ○ Part-time or hourly work 15 to 34 hours per week
- ○ Full-time work
- ○ On temporary leave (e.g. education leave, public service leave)
- ○ Decline to Answer

7) Do you work any night time shifts?
○ No
○ Yes
○ Decline to Answer

If yes…
7b) How frequently do you work at night? ____________________ nights/week

8) What type of residence do you currently live in?
○ Private home or apartment
○ Retirement home or senior complex
○ Nursing home
○ Personal care home (Adult foster home, assisted living)
○ Other: [please describe]___________________________________
○ Decline to Answer

9) Do you live alone?
○ Yes
○ No
○ Decline to Answer

If no…
9b) Who do you live with? (check all that apply)
□ Spouse
□ Child/children
□ Other family members
□ Friends
□ Nonrelatives
□ Declined to Answer

10) In general, would you say your health is:
_____ Excellent (1)
_____ Very Good (2)
_____ Good (3)
_____ Fair (4)
_____ Poor (5)

11) Place a mark on the line below to show the worst amount of pain you've felt in the last 24 hours:

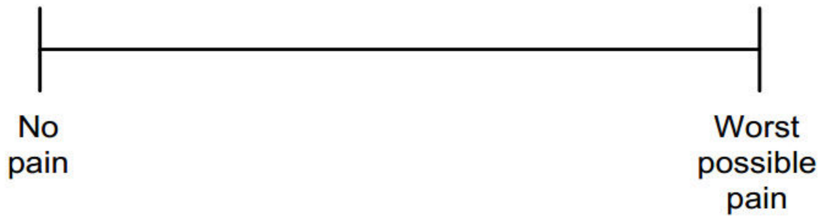

12) Have you ever been clinically diagnosed with a sleeping disorder?

○ Yes
○ No
○ Decline to Answer

If yes…

12b) Which of the following disorders have you been diagnosed with (please check all that apply)

□ Restless Leg Syndrome
□ Apnea
□ Insomnia
□ Periodic Leg Movement
□ Narcolepsy
□ Other:____

Sleep Diary: Study Day ______

COMPLETE THIS THE MORNING OF ______________________________

Date **Time**

1 What time did you try to go to sleep last night: ☐☐:☐☐ ○ am ○ pm

What time did you wake up this morning: ☐☐:☐☐ ○ am ○ pm

2 Place a mark on the line below to show the worst amount of pain you've felt in the last 24 hours:

No pain |————————————————| **Worst possible pain**

3 Have you taken your actigraph off in the last 24 hours? ○ Yes ○ No

If yes, what time did you take if off and put it back on?

	Time Off	Time On		Time Off	Time On
1)	☐☐:☐☐ ○ am ○ pm	☐☐:☐☐ ○ am ○ pm	2)	☐☐:☐☐ ○ am ○ pm	☐☐:☐☐ ○ am ○ pm
3)	☐☐:☐☐ ○ am ○ pm	☐☐:☐☐ ○ am ○ pm	4)	☐☐:☐☐ ○ am ○ pm	☐☐:☐☐ ○ am ○ pm

4 In the last 24 hours, have you taken a nap or fallen asleep during the day (5 minutes or longer)? ○ Yes ○No

If yes, what time did you take your nap(s)?

	From	Until		From	Until
1)	☐☐:☐☐ ○ am ○ pm	☐☐:☐☐ ○ am ○ pm	2)	☐☐:☐☐ ○ am ○ pm	☐☐:☐☐ ○ am ○ pm
3)	☐☐:☐☐ ○ am ○ pm	☐☐:☐☐ ○ am ○ pm	4)	☐☐:☐☐ ○ am ○ pm	☐☐:☐☐ ○ am ○ pm

5 In the last 24 hours, have you been able to get out of bed without the help of another person? ○Yes ○No

If not, what prevented you from being mobile? ______________________________

____

Sleep Diary Continued: Study Day ______

6 Would you consider your sleeping pattern during the last 24 hours while you wore the watch to be "normal" for you? ○ Yes ○ No

If no, on average how many times was your sleep interrupted last night? ____________

What caused your sleep to be interrupted? ______________________________________
____
____

THE FOLLOWING QUESTIONS ARE TO BE COMPLETED BY THE STUDY STAFF:

Is the participant in a shared room with another patient? ○ Yes ○ No

On which wrist does the participant have the actigraph? ○ Right ○ Left ○ The partipant is not wearing the actigraph

Discharge Form Packet

This standardized and validated test was completed at part of the discharge process. Please see the references for information on the forms:

Montreal Cognitive Assessment (MoCA)

Reference: Nasreddine, Z. S., Phillips, N. A., Bédirian, V., Charbonneau, S., Whitehead, V., Collin, I., et al. (2005). The Montreal Cognitive Assessment, MoCA: a brief screening tool for mild cognitive impairment. *Journal of the American Geriatrics Society*, *53*(4), 695–699. https://doi.org/10.1111/j.1532-5415.2005.53221.x
